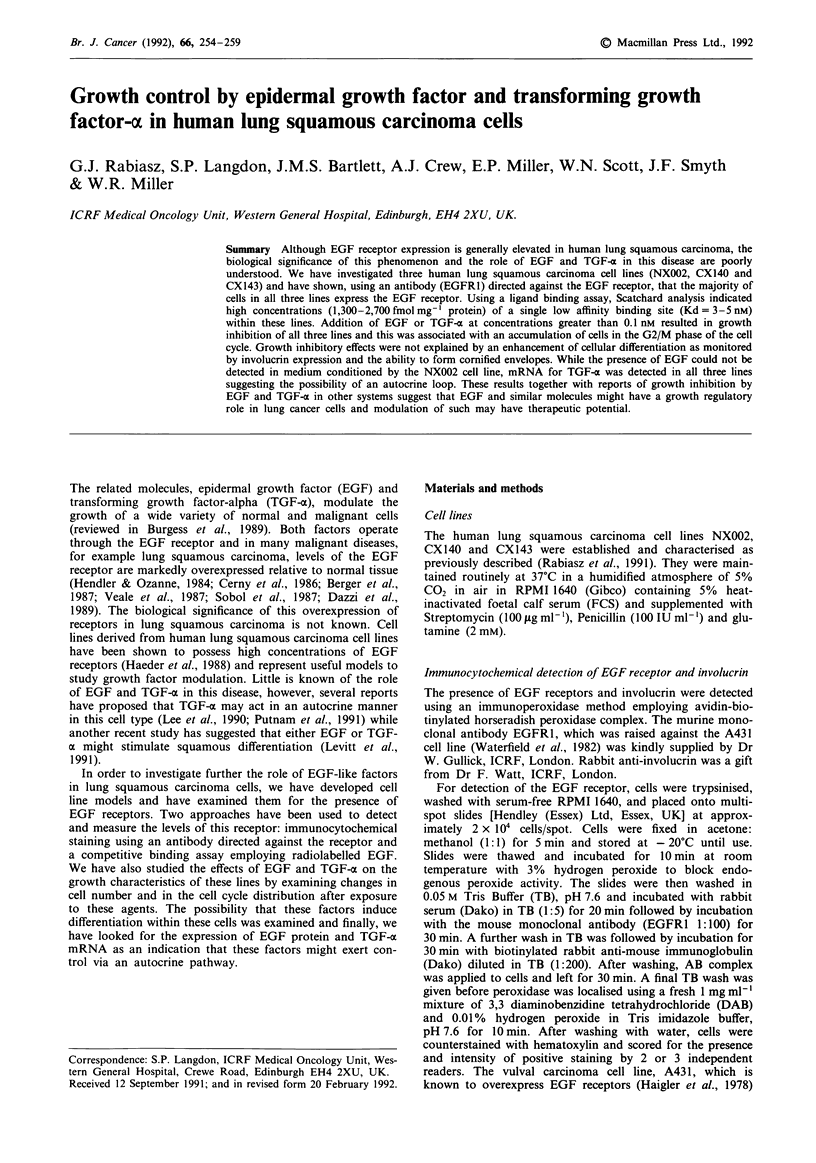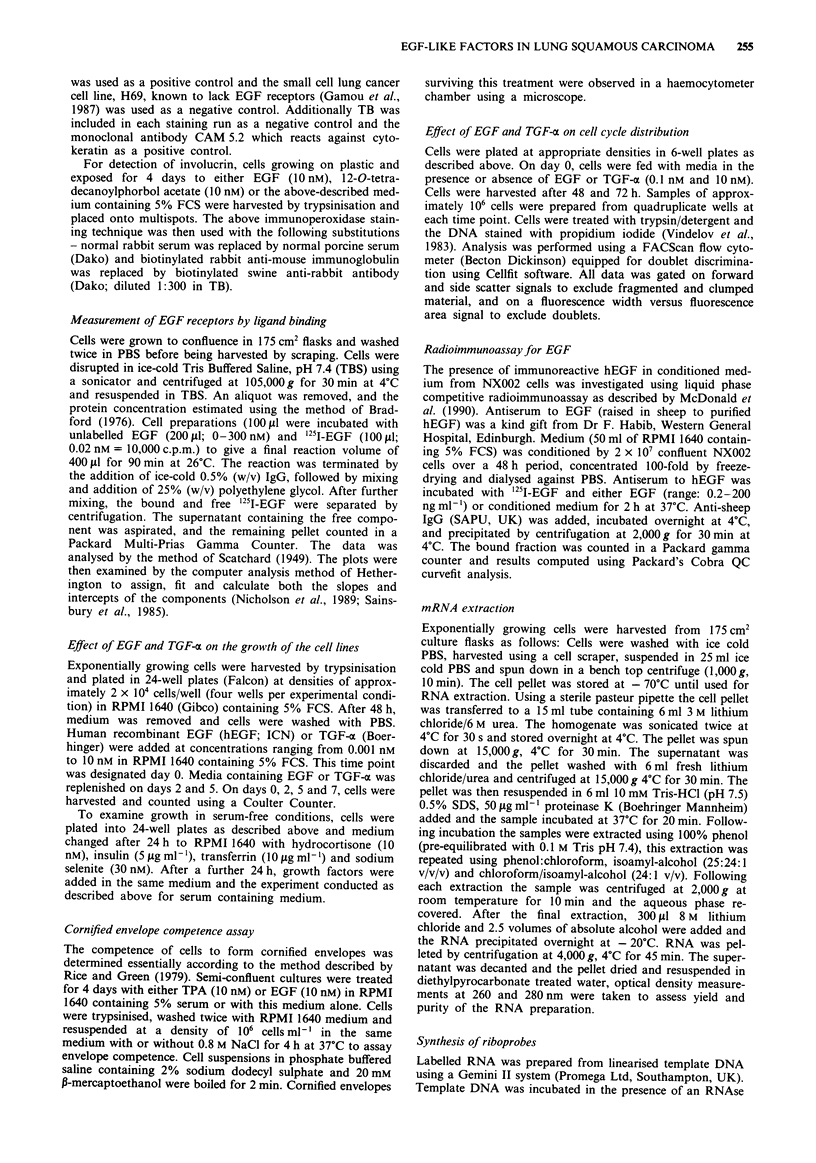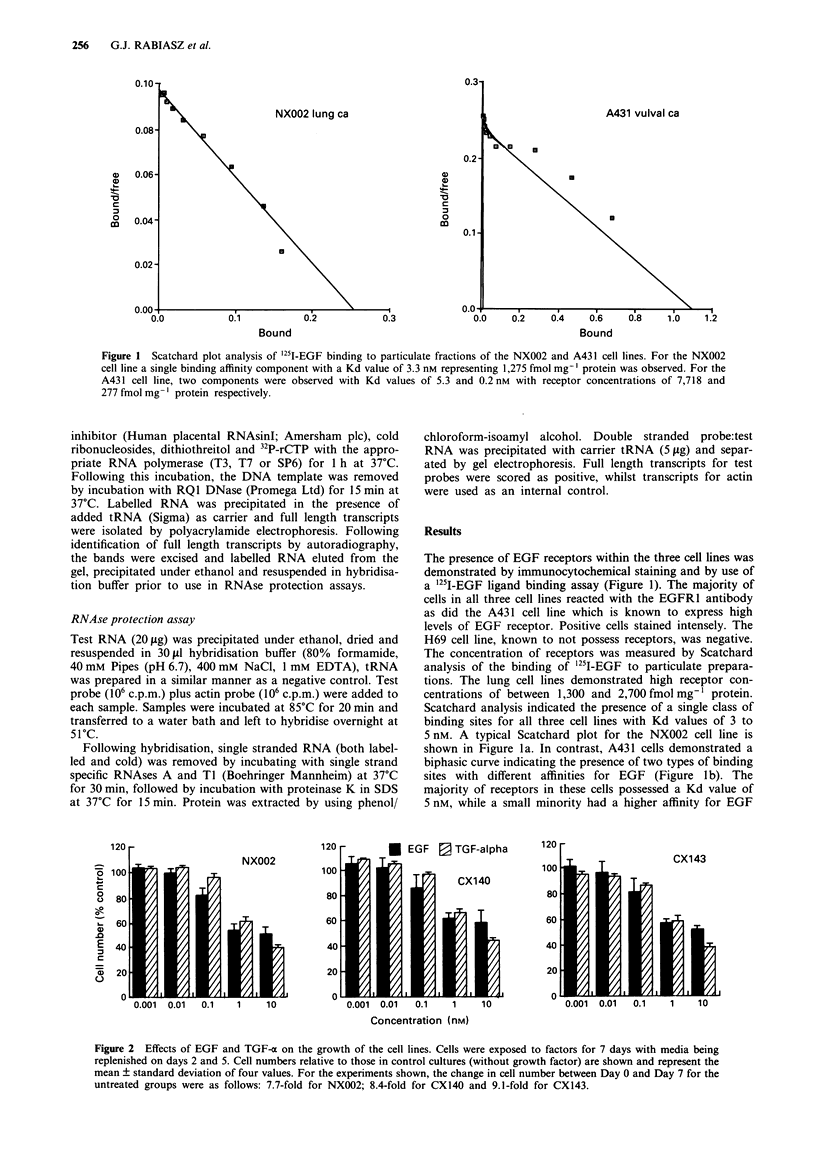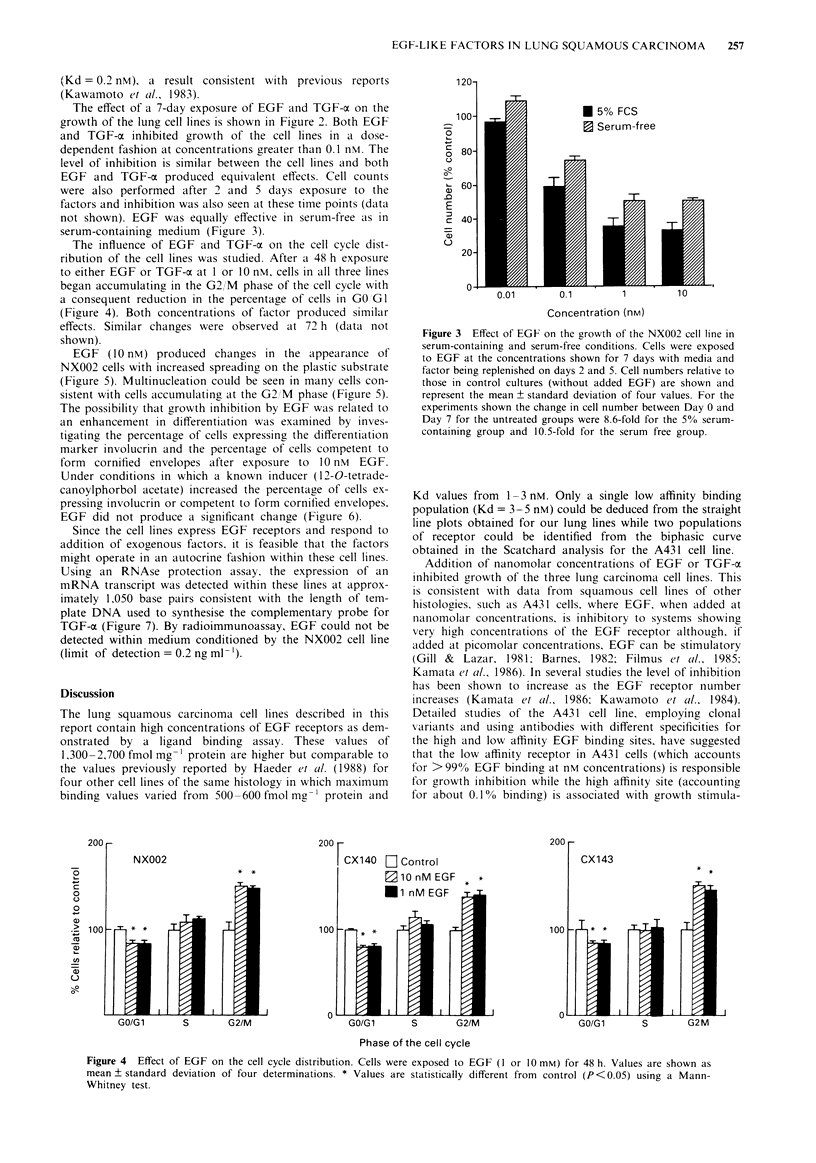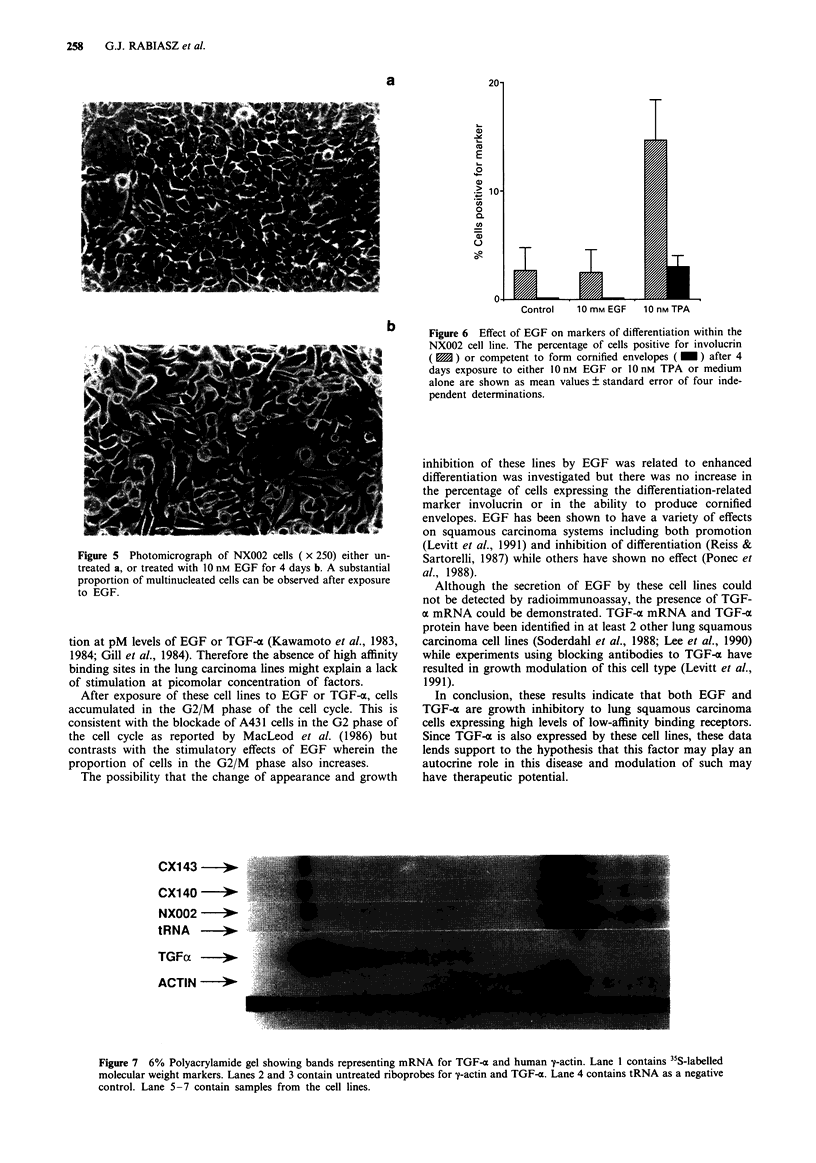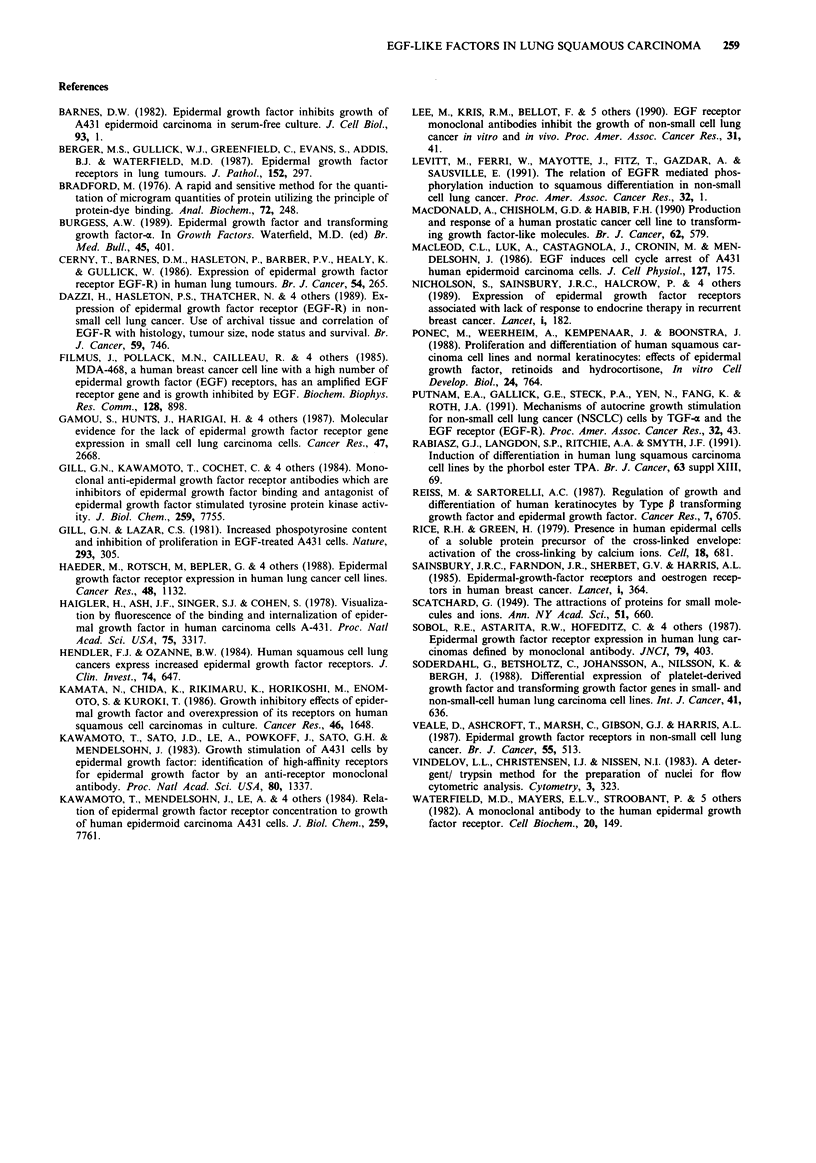# Growth control by epidermal growth factor and transforming growth factor-alpha in human lung squamous carcinoma cells.

**DOI:** 10.1038/bjc.1992.253

**Published:** 1992-08

**Authors:** G. J. Rabiasz, S. P. Langdon, J. M. Bartlett, A. J. Crew, E. P. Miller, W. N. Scott, J. F. Smyth, W. R. Miller

**Affiliations:** ICRF Medical Oncology Unit, Western General Hospital, Edinburgh, UK.

## Abstract

**Images:**


Br. J. Cancer (1992), 66, 254-259 © Macmillan Press Ltd., 1992

### Growth control by epidermal growth factor and transforming growth factor-α in human lung squamous carcinoma cells

G.J. Rabiasz, S.P. Langdon, J.M.S. Bartlett, A.J. Crew, E.P. Miller, W.N. Scott, J.F. Smyth & W.R. Miller

*ICRF Medical Oncology Unit, Western General Hospital, Edinburgh, EH4 2XU, UK.*

**Summary** Although EGF receptor expression is generally elevated in human lung squamous carcinoma, the biological significance of this phenomenon and the role of EGF and TGF-α in this disease are poorly understood. We have investigated three human lung squamous carcinoma cell lines (NX002, CX140 and CX143) and have shown, using an antibody (EGFR1) directed against the EGF receptor, that the majority of cells in all three lines express the EGF receptor. Using a ligand binding assay, Scatchard analysis indicated high concentrations (1,300–2,700 fmol $mg^{-1}$ protein) of a single low affinity binding site (Kd = 3–5 nM) within these lines. Addition of EGF or TGF-α at concentrations greater than 0.1 nM resulted in growth inhibition of all three lines and this was associated with an accumulation of cells in the G2/M phase of the cell cycle. Growth inhibitory effects were not explained by an enhancement of cellular differentiation as monitored by involucrin expression and the ability to form cornified envelopes. While the presence of EGF could not be detected in medium conditioned by the NX002 cell line, mRNA for TGF-α was detected in all three lines suggesting the possibility of an autocrine loop. These results together with reports of growth inhibition by EGF and TGF-α in other systems suggest that EGF and similar molecules might have a growth regulatory role in lung cancer cells and modulation of such may have therapeutic potential.

The related molecules, epidermal growth factor (EGF) and transforming growth factor-alpha (TGF-α), modulate the growth of a wide variety of normal and malignant cells (reviewed in Burgess *et al.*, 1989). Both factors operate through the EGF receptor and in many malignant diseases, for example lung squamous carcinoma, levels of the EGF receptor are markedly overexpressed relative to normal tissue (Hendler & Ozanne, 1984; Cerny *et al.*, 1986; Berger *et al.*, 1987; Veale *et al.*, 1987; Sobol *et al.*, 1987; Dazzi *et al.*, 1989). The biological significance of this overexpression of receptors in lung squamous carcinoma is not known. Cell lines derived from human lung squamous carcinoma cell lines have been shown to possess high concentrations of EGF receptors (Haeder *et al.*, 1988) and represent useful models to study growth factor modulation. Little is known of the role of EGF and TGF-α in this disease, however, several reports have proposed that TGF-α may act in an autocrine manner in this cell type (Lee *et al.*, 1990; Putnam *et al.*, 1991) while another recent study has suggested that either EGF or TGF-α might stimulate squamous differentiation (Levitt *et al.*, 1991).

In order to investigate further the role of EGF-like factors in lung squamous carcinoma cells, we have developed cell line models and have examined them for the presence of EGF receptors. Two approaches have been used to detect and measure the levels of this receptor: immunocytochemical staining using an antibody directed against the receptor and a competitive binding assay employing radiolabelled EGF. We have also studied the effects of EGF and TGF-α on the growth characteristics of these lines by examining changes in cell number and in the cell cycle distribution after exposure to these agents. The possibility that these factors induce differentiation within these cells was examined and finally, we have looked for the expression of EGF protein and TGF-α mRNA as an indication that these factors might exert control via an autocrine pathway.

Correspondence: S.P. Langdon, ICRF Medical Oncology Unit, Western General Hospital, Crewe Road, Edinburgh EH4 2XU, UK.
Received 12 September 1991; and in revised form 20 February 1992.

### Materials and methods

### *Cell lines*

The human lung squamous carcinoma cell lines NX002, CX140 and CX143 were established and characterised as previously described (Rabiasz *et al.*, 1991). They were maintained routinely at 37°C in a humidified atmosphere of 5% $CO_2$ in air in RPMI 1640 (Gibco) containing 5% heat-inactivated foetal calf serum (FCS) and supplemented with Streptomycin (100 μg $ml^{-1}$), Penicillin (100 IU $ml^{-1}$) and glutamine (2 mM).

### *Immunocytochemical detection of EGF receptor and involucrin*

The presence of EGF receptors and involucrin were detected using an immunoperoxidase method employing avidin-biotinylated horseradish peroxidase complex. The murine monoclonal antibody EGFR1, which was raised against the A431 cell line (Waterfield *et al.*, 1982) was kindly supplied by Dr W. Gullick, ICRF, London. Rabbit anti-involucrin was a gift from Dr F. Watt, ICRF, London.

For detection of the EGF receptor, cells were trypsinised, washed with serum-free RPMI 1640, and placed onto multispot slides [Hendley (Essex) Ltd, Essex, UK] at approximately $2 \times 10^4$ cells/spot. Cells were fixed in acetone: methanol (1:1) for 5 min and stored at − 20°C until use. Slides were thawed and incubated for 10 min at room temperature with 3% hydrogen peroxide to block endogenous peroxide activity. The slides were then washed in 0.05 M Tris Buffer (TB), pH 7.6 and incubated with rabbit serum (Dako) in TB (1:5) for 20 min followed by incubation with the mouse monoclonal antibody (EGFR1 1:100) for 30 min. A further wash in TB was followed by incubation for 30 min with biotinylated rabbit anti-mouse immunoglobulin (Dako) diluted in TB (1:200). After washing, AB complex was applied to cells and left for 30 min. A final TB wash was given before peroxidase was localised using a fresh 1 mg $ml^{-1}$ mixture of 3,3 diaminobenzidine tetrahydrochloride (DAB) and 0.01% hydrogen peroxide in Tris imidazole buffer, pH 7.6 for 10 min. After washing with water, cells were counterstained with hematoxylin and scored for the presence and intensity of positive staining by 2 or 3 independent readers. The vulval carcinoma cell line, A431, which is known to overexpress EGF receptors (Haigler *et al.*, 1978)

was used as a positive control and the small cell lung cancer cell line, H69, known to lack EGF receptors (Gamou *et al.*, 1987) was used as a negative control. Additionally TB was included in each staining run as a negative control and the monoclonal antibody CAM 5.2 which reacts against cytokeratin as a positive control.

For detection of involucrin, cells growing on plastic and exposed for 4 days to either EGF (10 nM), 12-*O*-tetradecanoylphorbol acetate (10 nM) or the above-described medium containing 5% FCS were harvested by trypsinisation and placed onto multispots. The above immunoperoxidase staining technique was then used with the following substitutions – normal rabbit serum was replaced by normal porcine serum (Dako) and biotinylated rabbit anti-mouse immunoglobulin was replaced by biotinylated swine anti-rabbit antibody (Dako; diluted 1:300 in TB).

*Measurement of EGF receptors by ligand binding*

Cells were grown to confluence in 175 $cm^2$ flasks and washed twice in PBS before being harvested by scraping. Cells were disrupted in ice-cold Tris Buffered Saline, pH 7.4 (TBS) using a sonicator and centrifuged at 105,000 *g* for 30 min at 4°C and resuspended in TBS. An aliquot was removed, and the protein concentration estimated using the method of Bradford (1976). Cell preparations (100 µl were incubated with unlabelled EGF (200 µl; 0–300 nM) and $^{125}$I-EGF (100 µl; 0.02 nM = 10,000 c.p.m.) to give a final reaction volume of 400 µl for 90 min at 26°C. The reaction was terminated by the addition of ice-cold 0.5% (w/v) IgG, followed by mixing and addition of 25% (w/v) polyethylene glycol. After further mixing, the bound and free $^{125}$I-EGF were separated by centrifugation. The supernatant containing the free component was aspirated, and the remaining pellet counted in a Packard Multi-Prias Gamma Counter. The data was analysed by the method of Scatchard (1949). The plots were then examined by the computer analysis method of Hetherington to assign, fit and calculate both the slopes and intercepts of the components (Nicholson *et al.*, 1989; Sainsbury *et al.*, 1985).

*Effect of EGF and TGF-α on the growth of the cell lines*

Exponentially growing cells were harvested by trypsinisation and plated in 24-well plates (Falcon) at densities of approximately $2 \times 10^4$ cells/well (four wells per experimental condition) in RPMI 1640 (Gibco) containing 5% FCS. After 48 h, medium was removed and cells were washed with PBS. Human recombinant EGF (hEGF; ICN) or TGF-α (Boerhinger) were added at concentrations ranging from 0.001 nM to 10 nM in RPMI 1640 containing 5% FCS. This time point was designated day 0. Media containing EGF or TGF-α was replenished on days 2 and 5. On days 0, 2, 5 and 7, cells were harvested and counted using a Coulter Counter.

To examine growth in serum-free conditions, cells were plated into 24-well plates as described above and medium changed after 24 h to RPMI 1640 with hydrocortisone (10 nM), insulin (5 µg $ml^{-1}$), transferrin (10 µg $ml^{-1}$) and sodium selenite (30 nM). After a further 24 h, growth factors were added in the same medium and the experiment conducted as described above for serum containing medium.

*Cornified envelope competence assay*

The competence of cells to form cornified envelopes was determined essentially according to the method described by Rice and Green (1979). Semi-confluent cultures were treated for 4 days with either TPA (10 nM) or EGF (10 nM) in RPMI 1640 containing 5% serum or with this medium alone. Cells were trypsinised, washed twice with RPMI 1640 medium and resuspended at a density of $10^6$ cells $ml^{-1}$ in the same medium with or without 0.8 M NaCl for 4 h at 37°C to assay envelope competence. Cell suspensions in phosphate buffered saline containing 2% sodium dodecyl sulphate and 20 mM β-mercaptoethanol were boiled for 2 min. Cornified envelopes surviving this treatment were observed in a haemocytometer chamber using a microscope.

*Effect of EGF and TGF-α on cell cycle distribution*

Cells were plated at appropriate densities in 6-well plates as described above. On day 0, cells were fed with media in the presence or absence of EGF or TGF-α (0.1 nM and 10 nM). Cells were harvested after 48 and 72 h. Samples of approximately $10^6$ cells were prepared from quadruplicate wells at each time point. Cells were treated with trypsin/detergent and the DNA stained with propidium iodide (Vindelov *et al.*, 1983). Analysis was performed using a FACScan flow cytometer (Becton Dickinson) equipped for doublet discrimination using Cellfit software. All data was gated on forward and side scatter signals to exclude fragmented and clumped material, and on a fluorescence width versus fluorescence area signal to exclude doublets.

*Radioimmunoassay for EGF*

The presence of immunoreactive hEGF in conditioned medium from NX002 cells was investigated using liquid phase competitive radioimmunoassay as described by McDonald *et al.* (1990). Antiserum to EGF (raised in sheep to purified hEGF) was a kind gift from Dr F. Habib, Western General Hospital, Edinburgh. Medium (50 ml of RPMI 1640 containing 5% FCS) was conditioned by $2 \times 10^7$ confluent NX002 cells over a 48 h period, concentrated 100-fold by freeze-drying and dialysed against PBS. Antiserum to hEGF was incubated with $^{125}$I-EGF and either EGF (range: 0.2–200 ng $ml^{-1}$) or conditioned medium for 2 h at 37°C. Anti-sheep IgG (SAPU, UK) was added, incubated overnight at 4°C, and precipitated by centrifugation at 2,000 *g* for 30 min at 4°C. The bound fraction was counted in a Packard gamma counter and results computed using Packard's Cobra QC curvefit analysis.

*mRNA extraction*

Exponentially growing cells were harvested from 175 $cm^2$ culture flasks as follows: Cells were washed with ice cold PBS, harvested using a cell scraper, suspended in 25 ml ice cold PBS and spun down in a bench top centrifuge (1,000 *g*, 10 min). The cell pellet was stored at − 70°C until used for RNA extraction. Using a sterile pasteur pipette the cell pellet was transferred to a 15 ml tube containing 6 ml 3 M lithium chloride/6 M urea. The homogenate was sonicated twice at 4°C for 30 s and stored overnight at 4°C. The pellet was spun down at 15,000 *g*, 4°C for 30 min. The supernatant was discarded and the pellet washed with 6 ml fresh lithium chloride/urea and centrifuged at 15,000 *g* 4°C for 30 min. The pellet was then resuspended in 6 ml 10 mM Tris-HCl (pH 7.5) 0.5% SDS, 50 µg $ml^{-1}$ proteinase K (Boehringer Mannheim) added and the sample incubated at 37°C for 20 min. Following incubation the samples were extracted using 100% phenol (pre-equilibrated with 0.1 M Tris pH 7.4), this extraction was repeated using phenol:chloroform, isoamyl-alcohol (25:24:1 v/v/v) and chloroform/isoamyl-alcohol (24:1 v/v). Following each extraction the sample was centrifuged at 2,000 *g* at room temperature for 10 min and the aqueous phase recovered. After the final extraction, 300 µl 8 M lithium chloride and 2.5 volumes of absolute alcohol were added and the RNA precipitated overnight at − 20°C. RNA was pelleted by centrifugation at 4,000 *g*, 4°C for 45 min. The supernatant was decanted and the pellet dried and resuspended in diethylpyrocarbonate treated water, optical density measurements at 260 and 280 nm were taken to assess yield and purity of the RNA preparation.

*Synthesis of riboprobes*

Labelled RNA was prepared from linearised template DNA using a Gemini II system (Promega Ltd, Southampton, UK). Template DNA was incubated in the presence of an RNAse

**Figure 1** Scatchard plot analysis of $^{125}$I-EGF binding to particulate fractions of the NX002 and A431 cell lines. For the NX002 cell line a single binding affinity component with a Kd value of 3.3 nM representing 1,275 fmol mg$^{-1}$ protein was observed. For the A431 cell line, two components were observed with Kd values of 5.3 and 0.2 nM with receptor concentrations of 7,718 and 277 fmol mg$^{-1}$ protein respectively.

inhibitor (Human placental RNAsinI; Amersham plc), cold ribonucleosides, dithiothreitol and $^{32}$P-rCTP with the appropriate RNA polymerase (T3, T7 or SP6) for 1 h at 37°C. Following this incubation, the DNA template was removed by incubation with RQ1 DNase (Promega Ltd) for 15 min at 37°C. Labelled RNA was precipitated in the presence of added tRNA (Sigma) as carrier and full length transcripts were isolated by polyacrylamide electrophoresis. Following identification of full length transcripts by autoradiography, the bands were excised and labelled RNA eluted from the gel, precipitated under ethanol and resuspended in hybridisation buffer prior to use in RNAse protection assays.

*RNAse protection assay*

Test RNA (20 μg) was precipitated under ethanol, dried and resuspended in 30 μl hybridisation buffer (80% formamide, 40 mM Pipes (pH 6.7), 400 mM NaCl, 1 mM EDTA), tRNA was prepared in a similar manner as a negative control. Test probe ($10^6$ c.p.m.) plus actin probe ($10^6$ c.p.m.) were added to each sample. Samples were incubated at 85°C for 20 min and transferred to a water bath and left to hybridise overnight at 51°C.

Following hybridisation, single stranded RNA (both labelled and cold) was removed by incubating with single strand specific RNAses A and T1 (Boehringer Mannheim) at 37°C for 30 min, followed by incubation with proteinase K in SDS at 37°C for 15 min. Protein was extracted by using phenol/chloroform-isoamyl alcohol. Double stranded probe:test RNA was precipitated with carrier tRNA (5 μg) and separated by gel electrophoresis. Full length transcripts for test probes were scored as positive, whilst transcripts for actin were used as an internal control.

### Results

The presence of EGF receptors within the three cell lines was demonstrated by immunocytochemical staining and by use of a $^{125}$I-EGF ligand binding assay (Figure 1). The majority of cells in all three cell lines reacted with the EGFR1 antibody as did the A431 cell line which is known to express high levels of EGF receptor. Positive cells stained intensely. The H69 cell line, known to not possess receptors, was negative. The concentration of receptors was measured by Scatchard analysis of the binding of $^{125}$I-EGF to particulate preparations. The lung cell lines demonstrated high receptor concentrations of between 1,300 and 2,700 fmol mg$^{-1}$ protein. Scatchard analysis indicated the presence of a single class of binding sites for all three cell lines with Kd values of 3 to 5 nM. A typical Scatchard plot for the NX002 cell line is shown in Figure 1a. In contrast, A431 cells demonstrated a biphasic curve indicating the presence of two types of binding sites with different affinities for EGF (Figure 1b). The majority of receptors in these cells possessed a Kd value of 5 nM, while a small minority had a higher affinity for EGF

**Figure 2** Effects of EGF and TGF-α on the growth of the cell lines. Cells were exposed to factors for 7 days with media being replenished on days 2 and 5. Cell numbers relative to those in control cultures (without growth factor) are shown and represent the mean ± standard deviation of four values. For the experiments shown, the change in cell number between Day 0 and Day 7 for the untreated groups were as follows: 7.7-fold for NX002; 8.4-fold for CX140 and 9.1-fold for CX143.

(Kd = 0.2 nM), a result consistent with previous reports (Kawamoto *et al.*, 1983).

The effect of a 7-day exposure of EGF and TGF-α on the growth of the lung cell lines is shown in Figure 2. Both EGF and TGF-α inhibited growth of the cell lines in a dose-dependent fashion at concentrations greater than 0.1 nM. The level of inhibition is similar between the cell lines and both EGF and TGF-α produced equivalent effects. Cell counts were also performed after 2 and 5 days exposure to the factors and inhibition was also seen at these time points (data not shown). EGF was equally effective in serum-free as in serum-containing medium (Figure 3).

The influence of EGF and TGF-α on the cell cycle distribution of the cell lines was studied. After a 48 h exposure to either EGF or TGF-α at 1 or 10 nM, cells in all three lines began accumulating in the G2/M phase of the cell cycle with a consequent reduction in the percentage of cells in G0/G1 (Figure 4). Both concentrations of factor produced similar effects. Similar changes were observed at 72 h (data not shown).

EGF (10 nM) produced changes in the appearance of NX002 cells with increased spreading on the plastic substrate (Figure 5). Multinucleation could be seen in many cells consistent with cells accumulating at the G2/M phase (Figure 5). The possibility that growth inhibition by EGF was related to an enhancement in differentiation was examined by investigating the percentage of cells expressing the differentiation marker involucrin and the percentage of cells competent to form cornified envelopes after exposure to 10 nM EGF. Under conditions in which a known inducer (12-*O*-tetradecanoylphorbol acetate) increased the percentage of cells expressing involucrin or competent to form cornified envelopes, EGF did not produce a significant change (Figure 6).

Since the cell lines express EGF receptors and respond to addition of exogenous factors, it is feasible that the factors might operate in an autocrine fashion within these cell lines. Using an RNAse protection assay, the expression of an mRNA transcript was detected within these lines at approximately 1,050 base pairs consistent with the length of template DNA used to synthesise the complementary probe for TGF-α (Figure 7). By radioimmunoassay, EGF could not be detected within medium conditioned by the NX002 cell line (limit of detection = 0.2 ng $ml^{-1}$).

### Discussion

The lung squamous carcinoma cell lines described in this report contain high concentrations of EGF receptors as demonstrated by a ligand binding assay. These values of 1,300–2,700 fmol $mg^{-1}$ protein are higher but comparable to the values previously reported by Haeder *et al.* (1988) for four other cell lines of the same histology in which maximum binding values varied from 500–600 fmol $mg^{-1}$ protein and

**Figure 3** Effect of EGF on the growth of the NX002 cell line in serum-containing and serum-free conditions. Cells were exposed to EGF at the concentrations shown for 7 days with media and factor being replenished on days 2 and 5. Cell numbers relative to those in control cultures (without added EGF) are shown and represent the mean ± standard deviation of four values. For the experiments shown the change in cell number between Day 0 and Day 7 for the untreated groups were 8.6-fold for the 5% serum-containing group and 10.5-fold for the serum free group.

Kd values from 1–3 nM. Only a single low affinity binding population (Kd = 3–5 nM) could be deduced from the straight line plots obtained for our lung lines while two populations of receptor could be identified from the biphasic curve obtained in the Scatchard analysis for the A431 cell line.

Addition of nanomolar concentrations of EGF or TGF-α inhibited growth of the three lung carcinoma cell lines. This is consistent with data from squamous cell lines of other histologies, such as A431 cells, where EGF, when added at nanomolar concentrations, is inhibitory to systems showing very high concentrations of the EGF receptor although, if added at picomolar concentrations, EGF can be stimulatory (Gill & Lazar, 1981; Barnes, 1982; Filmus *et al.*, 1985; Kamata *et al.*, 1986). In several studies the level of inhibition has been shown to increase as the EGF receptor number increases (Kamata *et al.*, 1986; Kawamoto *et al.*, 1984). Detailed studies of the A431 cell line, employing clonal variants and using antibodies with different specificities for the high and low affinity EGF binding sites, have suggested that the low affinity receptor in A431 cells (which accounts for >99% EGF binding at nM concentrations) is responsible for growth inhibition while the high affinity site (accounting for about 0.1% binding) is associated with growth stimula-

**Figure 4** Effect of EGF on the cell cycle distribution. Cells were exposed to EGF (1 or 10 mM) for 48 h. Values are shown as mean ± standard deviation of four determinations. * Values are statistically different from control ($P<0.05$) using a Mann-Whitney test.

**Figure 5** Photomicrograph of NX002 cells ( × 250) either untreated **a**, or treated with 10 nM EGF for 4 days **b**. A substantial proportion of multinucleated cells can be observed after exposure to EGF.

**Figure 6** Effect of EGF on markers of differentiation within the NX002 cell line. The percentage of cells positive for involucrin ( ▨ ) or competent to form cornified envelopes ( ■ ) after 4 days exposure to either 10 nM EGF or 10 nM TPA or medium alone are shown as mean values ± standard error of four independent determinations.

tion at pM levels of EGF or TGF-α (Kawamoto *et al.*, 1983, 1984; Gill *et al.*, 1984). Therefore the absence of high affinity binding sites in the lung carcinoma lines might explain a lack of stimulation at picomolar concentration of factors.

After exposure of these cell lines to EGF or TGF-α, cells accumulated in the G2/M phase of the cell cycle. This is consistent with the blockade of A431 cells in the G2 phase of the cell cycle as reported by MacLeod *et al.* (1986) but contrasts with the stimulatory effects of EGF wherein the proportion of cells in the G2/M phase also increases.

The possibility that the change of appearance and growth inhibition of these lines by EGF was related to enhanced differentiation was investigated but there was no increase in the percentage of cells expressing the differentiation-related marker involucrin or in the ability to produce cornified envelopes. EGF has been shown to have a variety of effects on squamous carcinoma systems including both promotion (Levitt *et al.*, 1991) and inhibition of differentiation (Reiss & Sartorelli, 1987) while others have shown no effect (Ponec *et al.*, 1988).

Although the secretion of EGF by these cell lines could not be detected by radioimmunoassay, the presence of TGF-α mRNA could be demonstrated. TGF-α mRNA and TGF-α protein have been identified in at least 2 other lung squamous carcinoma cell lines (Soderdahl *et al.*, 1988; Lee *et al.*, 1990) while experiments using blocking antibodies to TGF-α have resulted in growth modulation of this cell type (Levitt *et al.*, 1991).

In conclusion, these results indicate that both EGF and TGF-α are growth inhibitory to lung squamous carcinoma cells expressing high levels of low-affinity binding receptors. Since TGF-α is also expressed by these cell lines, these data lends support to the hypothesis that this factor may play an autocrine role in this disease and modulation of such may have therapeutic potential.

**Figure 7** 6% Polyacrylamide gel showing bands representing mRNA for TGF-α and human γ-actin. Lane 1 contains $^{35}$S-labelled molecular weight markers. Lanes 2 and 3 contain untreated riboprobes for γ-actin and TGF-α. Lane 4 contains tRNA as a negative control. Lane 5–7 contain samples from the cell lines.

### References

BARNES, D.W. (1982). Epidermal growth factor inhibits growth of A431 epidermoid carcinoma in serum-free culture. *J. Cell Biol.*, **93**, 1.

BERGER, M.S., GULLICK, W.J., GREENFIELD, C., EVANS, S., ADDIS, B.J. & WATERFIELD, M.D. (1987). Epidermal growth factor receptors in lung tumours. *J. Pathol.*, **152**, 297.

BRADFORD, M. (1976). A rapid and sensitive method for the quantitation of microgram quantities of protein utilizing the principle of protein-dye binding. *Anal. Biochem.*, **72**, 248.

BURGESS, A.W. (1989). Epidermal growth factor and transforming growth factor-α. In *Growth Factors*. Waterfield, M.D. (ed) *Br. Med. Bull.*, **45**, 401.

CERNY, T., BARNES, D.M., HASLETON, P., BARBER, P.V., HEALY, K. & GULLICK, W. (1986). Expression of epidermal growth factor receptor EGF-R) in human lung tumours. *Br. J. Cancer*, **54**, 265.

DAZZI, H., HASLETON, P.S., THATCHER, N. & 4 others (1989). Expression of epidermal growth factor receptor (EGF-R) in non-small cell lung cancer. Use of archival tissue and correlation of EGF-R with histology, tumour size, node status and survival. *Br. J. Cancer*, **59**, 746.

FILMUS, J., POLLACK, M.N., CAILLEAU, R. & 4 others (1985). MDA-468, a human breast cancer cell line with a high number of epidermal growth factor (EGF) receptors, has an amplified EGF receptor gene and is growth inhibited by EGF. *Biochem. Biophys. Res. Comm.*, **128**, 898.

GAMOU, S., HUNTS, J., HARIGAI, H. & 4 others (1987). Molecular evidence for the lack of epidermal growth factor receptor gene expression in small cell lung carcinoma cells. *Cancer Res.*, **47**, 2668.

GILL, G.N., KAWAMOTO, T., COCHET, C. & 4 others (1984). Monoclonal anti-epidermal growth factor receptor antibodies which are inhibitors of epidermal growth factor binding and antagonist of epidermal growth factor stimulated tyrosine protein kinase activity. *J. Biol. Chem.*, **259**, 7755.

GILL, G.N. & LAZAR, C.S. (1981). Increased phospotyrosine content and inhibition of proliferation in EGF-treated A431 cells. *Nature*, **293**, 305.

HAEDER, M., ROTSCH, M, BEPLER, G. & 4 others (1988). Epidermal growth factor receptor expression in human lung cancer cell lines. *Cancer Res.*, **48**, 1132.

HAIGLER, H., ASH, J.F., SINGER, S.J. & COHEN, S. (1978). Visualization by fluorescence of the binding and internalization of epidermal growth factor in human carcinoma cells A-431. *Proc. Natl Acad. Sci. USA*, **75**, 3317.

HENDLER, F.J. & OZANNE, B.W. (1984). Human squamous cell lung cancers express increased epidermal growth factor receptors. *J. Clin. Invest.*, **74**, 647.

KAMATA, N., CHIDA, K., RIKIMARU, K., HORIKOSHI, M., ENOMOTO, S. & KUROKI, T. (1986). Growth inhibitory effects of epidermal growth factor and overexpression of its receptors on human squamous cell carcinomas in culture. *Cancer Res.*, **46**, 1648.

KAWAMOTO, T., SATO, J.D., LE, A., POWKOFF, J., SATO, G.H. & MENDELSOHN, J. (1983). Growth stimulation of A431 cells by epidermal growth factor: identification of high-affinity receptors for epidermal growth factor by an anti-receptor monoclonal antibody. *Proc. Natl Acad. Sci. USA*, **80**, 1337.

KAWAMOTO, T., MENDELSOHN, J., LE, A. & 4 others (1984). Relation of epidermal growth factor receptor concentration to growth of human epidermoid carcinoma A431 cells. *J. Biol. Chem.*, **259**, 7761.

LEE, M., KRIS, R.M., BELLOT, F. & 5 others (1990). EGF receptor monoclonal antibodies inhibit the growth of non-small cell lung cancer *in vitro* and *in vivo*. *Proc. Amer. Assoc. Cancer Res.*, **31**, 41.

LEVITT, M., FERRI, W., MAYOTTE, J., FITZ, T., GAZDAR, A. & SAUSVILLE, E. (1991). The relation of EGFR mediated phosphorylation induction to squamous differentiation in non-small cell lung cancer. *Proc. Amer. Assoc. Cancer Res.*, **32**, 1.

MACDONALD, A., CHISHOLM, G.D. & HABIB, F.H. (1990) Production and response of a human prostatic cancer cell line to transforming growth factor-like molecules. *Br. J. Cancer*, **62**, 579.

MACLEOD, C.L., LUK, A., CASTAGNOLA, J., CRONIN, M. & MENDELSOHN, J. (1986). EGF induces cell cycle arrest of A431 human epidermoid carcinoma cells. *J. Cell Physiol.*, **127**, 175.

NICHOLSON, S., SAINSBURY, J.R.C., HALCROW, P. & 4 others (1989). Expression of epidermal growth factor receptors associated with lack of response to endocrine therapy in recurrent breast cancer. *Lancet*, **i**, 182.

PONEC, M., WEERHEIM, A., KEMPENAAR, J. & BOONSTRA, J. (1988). Proliferation and differentiation of human squamous carcinoma cell lines and normal keratinocytes: effects of epidermal growth factor, retinoids and hydrocortisone, *In vitro Cell Develop. Biol.*, **24**, 764.

PUTNAM, E.A., GALLICK, G.E., STECK, P.A., YEN, N., FANG, K. & ROTH, J.A. (1991). Mechanisms of autocrine growth stimulation for non-small cell lung cancer (NSCLC) cells by TGF-α and the EGF receptor (EGF-R). *Proc. Amer. Assoc. Cancer Res.*, **32**, 43.

RABIASZ, G.J., LANGDON, S.P., RITCHIE, A.A. & SMYTH, J.F. (1991). Induction of differentiation in human lung squamous carcinoma cell lines by the phorbol ester TPA. *Br. J. Cancer*, **63** suppl XIII, 69.

REISS, M. & SARTORELLI, A.C. (1987). Regulation of growth and differentiation of human keratinocytes by Type β transforming growth factor and epidermal growth factor. *Cancer Res.*, **7**, 6705.

RICE, R.H. & GREEN, H. (1979). Presence in human epidermal cells of a soluble protein precursor of the cross-linked envelope: activation of the cross-linking by calcium ions. *Cell*, **18**, 681.

SAINSBURY, J.R.C., FARNDON, J.R., SHERBET, G.V. & HARRIS, A.L. (1985). Epidermal-growth-factor receptors and oestrogen receptors in human breast cancer. *Lancet*, **i**, 364.

SCATCHARD, G. (1949). The attractions of proteins for small molecules and ions. *Ann. NY Acad. Sci.*, **51**, 660.

SOBOL, R.E., ASTARITA, R.W., HOFEDITZ, C. & 4 others (1987). Epidermal growth factor receptor expression in human lung carcinomas defined by monoclonal antibody. *JNCI*, **79**, 403.

SODERDAHL, G., BETSHOLTZ, C., JOHANSSON, A., NILSSON, K. & BERGH, J. (1988). Differential expression of platelet-derived growth factor and transforming growth factor genes in small- and non-small-cell human lung carcinoma cell lines. *Int. J. Cancer*, **41**, 636.

VEALE, D., ASHCROFT, T., MARSH, C., GIBSON, G.J. & HARRIS, A.L. (1987). Epidermal growth factor receptors in non-small cell lung cancer. *Br. J. Cancer*, **55**, 513.

VINDELOV, L.L., CHRISTENSEN, I.J. & NISSEN, N.I. (1983). A detergent/ trypsin method for the preparation of nuclei for flow cytometric analysis. *Cytometry*, **3**, 323.

WATERFIELD, M.D., MAYERS, E.L.V., STROOBANT, P. & 5 others (1982). A monoclonal antibody to the human epidermal growth factor receptor. *Cell Biochem.*, **20**, 149.